# Italian validation of the Children’s Shyness Questionnaire: Exploring associations between shyness and psychosocial functioning

**DOI:** 10.1371/journal.pone.0217722

**Published:** 2019-06-04

**Authors:** Valentina Spensieri, Rita Cerutti, Fabio Presaghi, Simone Amendola, W. Ray Crozier

**Affiliations:** 1 Department of Dynamic and Clinic Psychology, Sapienza University, Rome, Italy; 2 Department of Psychology of Developmental and Social Processes, Sapienza University, Rome, Italy; 3 School of Social Sciences, Cardiff University, Cardiff, United Kingdom; Universita Cattolica del Sacro Cuore Sede di Roma, ITALY

## Abstract

**Background:**

In recent years, researchers have begun to explore the implications of shyness for the psychosocial wellbeing of children and adolescents, exploring its association with internalizing problems. Research in an Italian context is hindered by the lack of a validated self-report measure of shyness. We report two studies aimed to assess the psychometric properties of an Italian translation of the Children’s Shyness Questionnaire (CSQ-it) and investigate its correlations with convergent and divergent constructs. The first study aimed to examine associations between CSQ-it and self-report measures of anxiety and somatic symptoms and attachment with parents and peers. The second study aimed to investigate its relations to internet addiction.

**Methods:**

The self-report measures were completed by 550 participants in the first study and 131 participants in the second study. Parents provided information on their child’s problems. Psychometric properties were assessed by Cronbach’s alpha in both studies and by exploratory and confirmatory factor analysis in Study 1. The relations between shyness and measures of internalizing problems and attachments were analyzed by correlational methods. In Study 2 a moderated mediation model tested the hypothesis that the relationship between shyness and internet addiction is mediated by somatic symptoms and that shyness moderates the relationship between somatic symptoms and internet addiction.

**Results:**

The reliability and validity of the Italian Version of the Children’s Shyness Questionnaire were satisfactory. Results from confirmatory factor analyses confirmed the single-factor model of the questionnaire previously identified in North American and Chinese studies. There were significant correlations between shyness, anxious and somatic symptomatology, impaired psychosocial functioning and specific components of attachment relationships. In Study 2 the indirect effect of shyness on internet addiction through somatic symptoms was significant as well as significantly moderated for high shyness scores but not for low levels of shyness.

**Conclusion:**

To our knowledge this is the first study that explored the psychometric proprieties of the Children’s Shyness Questionnaire in the Italian context. Findings demonstrated that this self-reported measure of shyness has sound psychometric properties and can be used as a sensitive and appropriate instrument for the assessment of shyness in children and adolescents.

## Introduction

Psychologists define shyness as a temperament or personality trait that is characterized by wariness and anxiety in the face of social novelty and perceived social evaluation, reticence in social situations, and embarrassment and self-consciousness in situations where shy individuals perceive themselves as being, or likely to be, socially evaluated [1].

This definition distinguishes it from related constructs such as behavioral inhibition and social withdrawal. Research suggests that behavioral inhibition represents a risk factor for the development of social anxiety [2,3]. However, in contrast to shyness, behavioral inhibition describes a temperamental trait that refers to avoiding interaction with social and non-social novel stimuli that are experienced as a source of anxiety or distress [4]. On the other hand, social withdrawal can be interpreted as an umbrella term describing a behavioral prototype (solitude in one form or another) deriving from different causes (both internal and external). Further, it can result from preference for solitude (i.e., internal causes), but also from social rejection (i.e. external cause).

There is evidence that behavioral inhibition predicts shyness as well as anxiety symptoms and disorders [5]. It has been shown that highly reactive (inhibited) 4-month-olds are more likely than their non-highly reactive peers to exhibit anxiety symptoms at age 7 [6]. Furthermore, adults with social anxiety disorder tend to report having been shy and socially reserved in childhood [7].Shyness appears to be characterized by high social approach and high social avoidance motivation. Specifically, shy children desire relationships with their peers but they experience uncomfortable feelings because of their inaction, self-consciousness and heightened physiological reactions.

### Shyness and somatic symptoms

Chung and Evans [8] have shown that shy children suffer more than their peers from health problems such as gastrointestinal disorders that lead to more frequent absences from school. They also experience more general difficulties at school. The authors hypothesized that somatic symptoms could reflect altered physiological functioning, with a hypersecretion of cortisol that suppresses the immune system. Furthermore, frequent school absences could exacerbate feelings of anxiety and discomfort, reinforcing the withdrawn behavior of shy children. The link between shyness and somatic symptoms was identified in a recent investigation by Piko, Varga and Mellor [9], drawing upon a sample of 490 Hungarian adolescents. Shyness, particularly when combined with social withdrawal, was associated with somatic disorders, loneliness and isolation, neuroticism, anxiety and depression. Several studies have shown that nervousness, tension and worrying may contribute to the onset and persistence of somatic symptoms [10,11]. Since these traits are characteristic of shy individuals, it is reasonable to expect that shyness is associated with the development of somatic symptomatology [11]. A lack of confidence in their skills can lead shy children to disengage from school activities, resulting in a negative evaluation of their performance by teachers and low academic performance [12]. Impaired school functioning, along with their difficulties in relationships with teachers and peers, may contribute to generating conflict situations that cause profound discomfort and social anxiety symptoms, triggering physiological responses and somatic symptoms [13,14].

### Shyness and social relationships

Early social interactions between children and caregivers are particularly important for child emotional development [15,16], providing information on how the child’s mental representations of self and others develop and influence the quality of future relationships [17]. The importance of first attachment relationships is related not only to the regulation of interpersonal functioning, but also to the development of a positive, coherent and well-organized self-structure. Several studies have highlighted that children with a secure attachment have better social and behavioral skills, experience fewer behavioral difficulties and enjoy more positive interactions with both peers and adults compared to children with insecure attachment [18]. In contrast, children with insecure attachments experience feelings of low self-efficacy and low self-esteem and perceive the world as an unpredictable and frightening place. In this way, they are more likely to develop inhibited behaviors in unfamiliar situations.

Empirical studies have identified significant associations between insecure attachment, shyness and behavioral inhibition [19,20]. Nachmias and colleagues [19] explored the role of attachment in the relationship between behavioral inhibition and stress reactivity in a sample of 78 18-monthold children and their mothers. Findings supported the hypothesis that attachment security can moderate the effect of behavioral inhibition on the activation of the hypothalamic-pituitary-adrenocortical system which secretes cortisol, a stress hormone involved in response to physical and/or emotional stress. Similarly, Spangler and Schieche [20] reported that the activation of adrenocortical system was most prominent in infants with insecure attachment and high behavioural inhibition, demonstrating the role of secure attachment relationship as a social buffer against less adaptive temperamental dispositions.

Shyness and inhibition in childhood are associated with impairment of social functioning, including peer rejection, isolation and school failure [21]. Inhibited children are often seen as less socially competent than their peers, with increased feelings of loneliness, dissatisfaction, negative self-perception, anxiety and depression [22,23].

Chan [24] found that among a sample of 326 Chinese children and adolescents, shy teenagers tended to feel uncomfortable while interacting with others because their low self-esteem was associated with the belief that others held high expectations of them. For this reason, the fear of disappointing others, being rejected or appearing foolish, encouraged shy children to withdraw from social interactions in order to avoid a negative evaluation and the consequent social rejection. In this way, a self-perpetuation cycle is created in which the self-esteem of shy children continually diminishes, further limiting their ability to engage in social relationships [24].

### Shyness and internet addiction

In recent decades there has been increasing interest in internet use among children and adolescents worldwide [13,25,26,27]. Studies of the use of the internet in adolescence have mainly focused on the consequences of its overuse, highlighting how the internet can have harmful effects on adolescent psychosocial well-being, both at school and at home [28,29,30]. However, adolescents who use the internet should not be categorized as a homogeneous group, and studies of the impact of the internet on psychosocial functioning should take individual differences into account [29]. In recent years, there has been a growing interest in the study of personal characteristics, such as shyness and loneliness, which may predispose adolescents to the development of cognitive and behavioral patterns related to internet use [25].

According to self-presentation theory [31], shy individuals, like all individuals, try to control their self-image and any information that relates to their identity, in the attempt to present a positive image to others, with the aim of obtaining social approval and minimizing disapproval [32]. However, unlike their non-shy peers, shy people tend to be less confident and competent, doubting their ability to create positive impressions [33]. They typically have fewer friends, experience less satisfactory social interactions and receive less social support [34]. For these reasons the internet can provide an environment that facilitates interaction, as it allows shy people a greater opportunity to manage the impression they make on others.

With regard to negative behaviors related to the excessive use of new technologies, Chak and Leung [35] reported a significant association between high levels of shyness in adolescents and an increased probability of internet addiction. Similar findings were reported by Roberts, Smith and Pollock [34] who demonstrated the tendency of shy people, to feel braver during online conversations than in face-to-face conversations, and their more frequent use of the internet as a social medium in order to satisfy their need to be in relationship with others. These results support the notion that the internet provides an alternative environment for the fulfilment of emotional and relational needs. However, this pattern could trigger a cycle of addiction, given that the longer children and adolescents spend online, the less they are available for face-to-face interactions, leading to excessive and uncontrolled use of the internet and a consequent increase in shyness and social isolation [29]. Research into self-reported shyness and the internet use of young people in Italy requires a measure of shyness that is validated for the Italian population. This study assesses the psychometric properties of one widely used measure and considers its relation to internet use.

### Methodological issues in measuring shyness in children

A substantial body of research has investigated shyness and its correlates among school-age children. Shyness has been assessed in various ways, through observations of younger children while they are interacting with a stranger [36], during free play with unfamiliar peers [37] or in kindergarten [38]. These methods are less appropriate for older children and research with school-age children has relied upon parent and teacher ratings of shyness or their responses to questionnaires such as the Child Social Preference Scale [39], an 11-item scale with items referring to a child’s typical play behavior, and the Children’s Behavior Questionnaire (CBQ) [40]. There is a dearth of self-report questionnaires for shyness, although a measure for adults, the Revised Cheek and Buss Shyness scale [41,42,43] has been widely used. It is important to assess children’s self-perceptions because shyness is not merely a matter of observed reticence and anxiousness but involves the child’s feelings of self-consciousness and fear of negative self-evaluation. Reticence is not necessarily due to shyness and shy children can have shy thoughts and feelings without visible manifestation.

Crozier [44] developed the Children’s Shyness Questionnaire (CSQ), a self-report questionnaire comprising 26 items based on interviews with children about their understanding of shyness and the situations associated with it. The interviews and subsequent questionnaire were targeted at the age range 9 to 12 years. The questionnaire was completed by a sample of 137 British children within this age range and showed satisfactory reliability (Cronbach’s alpha = 0.82). In a second sample comprising 232 children, alpha was also 0.82. In both samples there were significant negative correlations between shyness and various measures of self-esteem, consistent with findings in the literature. Three Canadian studies have incorporated the CSQ. Findlay, Coplan and Bowker [45] reported alpha of 0.77; Coplan, Rose-Krasnor, Weeks, Kingsbury, Kingsbury and Bullock [46] reported a coefficient of 0.87. Spooner, Evans and Santos [47] reported alpha of 0.88; a supplementary factor analysis of the CSQ identified two factors: one that appeared to tap self-conscious shyness and embarrassment (‘I am easily embarrassed’) and one that tapped more social aspects of shyness and sociability (‘I am usually quiet when with others’); Deng, Liu, Coplan, Chen, Li and Sang [48] translated the scale into Chinese and submitted this version to confirmatory factor analysis. The data fitted a single-factor model; six items had loadings lower than .30 so they continued with a 19-item version with alpha = 0.83.

The CSQ was designed to produce a range of shyness scores. Specifically, each item is scored 0,1 or 2, and a total score is calculated for the set of items by adding the scores for each item. In line with Crozier’s suggestion that high levels of shyness could be defined in terms of scores more than one standard deviation above the mean. The use of a cut-off score is useful to explore whether high levels of shyness impact children’s psychological and physical wellbeing.

### Current study

In order to validate the Italian Version of the CSQ (CSQ-it), two separate studies were carried out.

The aim of the first study was to develop an Italian language version of the CSQ, examine its psychometric properties and its correlations with relevant variables. This will extend the use of the scale to a European language other than English and will facilitate research into children’s shyness among the Italian population. The aim of the second study was to administer the CSQ to a fresh sample and investigate the relationship between children’s shyness and their use of the internet. We hypothesized that this association would be mediated by levels of somatic symptoms, based on evidence demonstrating that shyness may lead to excessive use of internet [35], that is in turn associated with somatic symptomatology [49].

## Study 1: Validation of the CSQ-it

The CSQ was translated into Italian and its reliability and factorial structure were examined. The study tested relationships between the CSQ and four relevant variables drawn from previous research into children’s shyness: somatic symptomatology; anxiety; difficulties in physical functioning; attachment relationships to both parents and peers.

## Method

### Participants

Five hundred and fifty Italian schoolchildren were recruited from public schools in Italy and involved in the present study. Based on the pre-analysis data screening, a total of 82 participants were excluded from the dataset due to missing and outlier responses (>10%). In addition to missing values, exclusion criteria for participation in the current study included the presence of a diagnosed psychiatric illness, history of a significant neurological illness or brain injury and the use of medications that could affect study outcomes. The final sample comprised 468 schoolchildren (52.1% males) aged 8 to 12 years (M = 9.4, SD = .87). All participants were Caucasian.

### Procedure

A written informed consent was obtained from all schoolchildren and their parents before their enrolment in the study. All participants included in the final sample (N = 468) completed the questionnaire battery, administered in written form, in the classroom during school time. The collective administration took approximately 30–45 minutes. Anonymity of participants was ensured. This study was approved by the Ethics Committee of the Department of Dynamic and Clinical Psychology, Sapienza University of Rome.

### Measures

#### Assessment schedule of children’s health

Parents were asked to report upon their child’s physical and mental health status by completing a checklist of medical illnesses and/ or existing diagnoses. Further, they had to indicate if their child was undergoing any pharmacological or psychological therapy. Parents received the questionnaire via their children, with the request to complete it at home and return materials in a sealed envelope. Anonymity of participants was ensured.

#### Shyness

Shyness was assessed by means of the Children’s Shyness Questionnaire (CSQ) [44, 48]. The CSQ comprises 26 items describing the emotional and behavioral aspects of shyness (e.g., Item 1: ‘‘I find it hard to talk to someone I don’t know”; Item 6:‘‘I feel shy when I have to read aloud in front of the class”). For this age range a simple response format was required, so for each question children were asked to tick one of three boxes: “Yes”, “No”, or “Don’t know”. The questionnaire was translated into Italian using the translation–back-translation method, with the permission of the questionnaire’s author. The factor structure and psychometric properties of the CSQ-it are reported in the Results section.

#### Anxiety

In order to investigate the presence and severity of anxiety disorders in children, the Screen for Child Anxiety-Related Emotional Disorders (SCARED) [50, 51, 52] was used. The SCARED consists of 41 items that evaluate various types of disorders, according to the nosography of the DSM-IV-TR [53]: somatic / panic symptoms, generalized anxiety, separation anxiety, social anxiety and school phobia. Children were asked to indicate how much each item described their feelings and behaviors related to the last three months using a 3-point Likert response scale, from 0 “Not true or hardly ever true” to 2 “Very true or often true”. Previous research has reported that the SCARED has good internal consistency (α = 0.74 to 0.93) and good 5-week test-retest reliability [50]. In the present study, Cronbach’s alpha for the SCARED was .92. For the “Somatic/panic symptoms” subscale it was .84, for the “Generalized anxiety” subscale .73, for the “Separation anxiety” subscale .75, for the “Social anxiety” subscale .76 and for the “School phobia” subscale” .58.

#### Somatic symptoms

The Children’s Somatization Inventory-24 Child Version (CSI-24) [54,55] assessed children’s perceptions of somatic symptoms. The self-report questionnaire was translated into Italian using the translation–back-translation method, with the approval of the author. The CSI-24 comprises 24 items with a 5-point Likert response scale (0 “Not at all”; 1 “A little”; 2 “Somewhat”; 3 “A lot” and 4 “A whole lot”). Satisfactory reliability and validity of the CSI-24 has been established. In healthy pediatric samples, internal consistency (i.e., Cronbach’s alpha) of the CSI-24 was .87 [55]. Cronbach’s alpha in the current study was .89.

#### Child impairment

The Functional Disability Inventory (FDI) [56] was used to explore children’s difficulties in physical and psychosocial functioning due to their physical health. The FDI was translated into Italian with the translation–back-translation method and approved by the Inventory’s author. Functional difficulties are rated in 15 items with a 5 point Likert response scale, from 0 “No trouble” to 4 “Impossible”, concerning perceptions of activity limitations during the past two weeks, including the performance of daily activities at home, school, recreation, and social situations. The FDI has good internal consistency and 3-month test-retest reliability estimates exceeding 0.60 for patients with chronic abdominal pain [56]. In the current study Cronbach’s alpha was .85.

#### Attachment to parents and peers

The Inventory of Parent and Peer Attachment (IPPA) [57,58,59] revised version for children (IPPA-R) [60] was used to explore children’s perceptions of attachment to their parents and peers. The questionnaire assesses the positive and negative affective component and the cognitive dimensions of the relationships that children have with parents and close friends. The IPPA includes two scales, one relating to parental attachment (IPPA-Parents) that comprises 28 items, and the other related to peer attachment (IPPA-Peers) consisting of 25 items. The response scale of both IPPA Parents and the IPPA Peers is a 5-point Likert scale (1 “Almost never or never true”; 2 “Not very often true”; 3 “Sometimes true”; 4 “Often true” and 5 “Almost always or always true”). Studies of the validity of constructs on both IPPA Parents and IPPA Peers scales have identified the presence of three dimensions, named Trust, Communication and Alienation. The IPPA shows good internal consistency with values between 0.72 and 0.91[58] and good test-retest reliability, with correlation coefficients of r = 0.86 for attachment to peers and r = 0.93 for attachment to parents.

In the current study the IPPA Parents scale has Cronbach’s alpha of .83 for the total score, .66 for the “Trust” dimension, .66 for the “Communication” dimension and .79 for the “Alienation” dimension. Regarding the IPPA Peers reliability, Cronbach’s alpha was .82 for the total score, .77 for the “Trust” dimension, .83 for the “Communication” dimension and .68 for the “Alienation” dimension.

### Analysis strategy

Exploratory Factor Analysis (EFA) was used to investigate the dimensionality of the Italian version of the CSQ (CSQ-it). Because the CSQ items have a categorical response scale with three values (False = 0; Not false nor true = 1; True = 2) we factor analyzed the polychoric correlation matrix using the statistical package “psych” by Revelle [61] within the statistical environment of R software [62]. EFA was necessary as past studies have shown that not all items from the CSQ report satisfactory factors loadings [44,46,48]. Confirmatory Factor analysis was utilized to estimate the statistical fit of the emerging factor model of the CSQ-it. In this case, the statistical package “lavaan” developed by Rossell [63] within the R environment was used. In order to test the exploratory and confirmatory factor structure of the CSQ-it on independent samples, the total sample was randomly split into two independent samples: the first sample (N = 234) was used for the EFA; the second for the CFA (N = 234). To determine whether the sample size of the two random split samples was large enough for factor analysis, we ran a power analysis for Structural Equation Models based on the RMSEA method proposed by MacCallum, Browne and Sugawara [64] using the R package “WebPower” by Zhang and Yuan [65]. Results showed that for a RMSEA of .08 (the typical cutoff below which the model fit is considered adequate), a critical alpha of .05, and a model with 152 d.f. (one factor model with 19 observed variables) and a sample size of N = 200, the estimate’s statistical power is approximately 1.00. Considering a more stringent scenario and assuming a RMSEA of .05 (smaller than the accepted cutoff), statistical power was 0.97. Based on the power analysis, the minimum sample size to detect an RMSEA of .08, given a power of 0.80 and the model parameter indicated above, was approximately N = 51. By increasing the power to 0.90, the optimal sample size was approximately N = 62. If we consider an expected RMSEA of 0.05 and a power of 0.90, then we obtain an optimal sample size of N = 156. Based on these calculations, the size of the two random split samples seems to be adequate.

Furthermore, to investigate the predictive validity of CSQ-it we derived a cut-off score by considering the mean plus one standard deviation as the cut-off point. We compared children above the cut-off score with those below the cut-off score on all psychopathological dimensions assessed.

## Results

### Psychometric properties of the Italian version of the children’s shyness questionnaire

An Exploratory Factor Analysis with polychoric correlations was performed on the 26 CSQ-it items. By inspecting the eigenvalues sequence of the first solution (eigenvalues: 5.39; 1.75; 0.85), two factors reported eigenvalues greater than 1, and only the first factor explained a percentage of total variance greater than 10% (specifically 20.7%). The parallel analysis indicated that at least seven factors exceeded the factors obtained from randomly simulated polychoric correlation matrices. In line with Crozier’s [44] and others’[46,48], we selected the first factor. In line with previous research [44,46,48], we identified six items with factor loadings lower than .35 (item 3; item 9; item 15; item 16; item 18; item 23) that had been excluded from further analyses. The final version of the CSQ-it is unidimensional, comprising 19 items with factor loadings exceeding .35; it explained approximately 27% of the total correlations and demonstrated good internal consistency (Cronbach α = 0.80).

#### Confirmatory factor analysis of CSQ-it

On the second independent sample we conducted Confirmatory Factor Analysis (CFA) using the lavaan package [45] in R statistical software [62] to evaluate the fit of the single-factor model of the CSQ-it. Given that the response scale is a three-step categorical scale, we used a Maximum Likelihood estimator with robust standard errors. Results indicated that, overall, the unifactor model fit is satisfactory (Satorra-Bentler scaled χ^2^ = 24991, df = 152, p < .01; RMSEA = .05, 90% C.I.: .04 - .06; SRMR = 0.06). Only two fit indices were unsatisfactory: CFI = .85 and NNFI = .83. Table 1 shows the standardized factor loadings for the CSQ-it. For this sample, the reliability of the Shyness factor was good (Cronbach α = 0.81).

**Table 1:** Standardized factor loadings of the 19 items of the CSQ-it.

Item	Factor loading	s.e.
CSQ1 I find it hard to talk to someone I don’t know.	0.472	0.057
CSQ2 I am easily embarrassed.	0.599	0.048
CSQ4 Do you blush when people sing ‘Happy Birthday’ to you?	0.417	0.057
CSQ5 I feel nervous when I am with important people.	0.429	0.059
CSQ6 I feel shy when I have to read aloud in front of the class.	0.458	0.059
CSQ7 I feel nervous about joining a new class.	0.333	0.066
CSQ8 I go red when someone teases me.	0.391	0.064
CSQ11 I am usually shy in a group of people.	0.566	0.048
CSQ12 I feel shy when I am the centre of attention.	0.499	0.056
CSQ13 Do you blush a lot?	0.423	0.062
CSQ14 I feel shy when the Head Teacher speaks to me.	0.399	0.059
CSQ17 I would be embarrassed if the teacher put me in the front row on stage.	0.450	0.058
CSQ19 I go red when the teacher praises my work.	0.429	0.063
CSQ20 I feel shy when I have to go into a room full of people.	0.455	0.060
CSQ21 Are you embarrassed when your friends look at photos of you when you were little?	0.185	0.069
CSQ22 Would you be too shy to ask someone to sponsor you for a good cause?	0.407	0.063
CSQ24 I usually talk to only one or two close friends.	0.147	0.074
CSQ25 I am usually shy when I meet girls (boys).	0.535	0.051
CSQ26 I go red whenever I have to speak to a girl (boy) of my age.	0.412	0.056

#### Cut-off scores for CSQ-it

The total sample (N = 468) was divided based on the cut-off score of the CSQ in order to investigate whether shyer children were more likely to report higher levels of somatic and anxious symptoms as well as less attachment to parents and peer. In accordance with Crozier’s suggestion [44] regarding the cut-off score of the instrument (± 1 standard deviation above the mean, M = 16.5, SD = 8.0, cut off value = 24.5), shy children (N = 71) were distinguished from non-shy (N = 397). Table 2 shows the descriptive statistics carried out exclusively on the sample of shy children (N = 71). Analysis of variance showed no statistically significant differences between males (N = 31) and females (N = 40) on any of the attachment and somatic symptoms measures.

**Table 2:** Descriptive statistics of shy children.

	*Males*		*Females*		*Total*	
	Mean	SD	Mean	SD	Mean	SD
**CSI-24** ^**a**^	4.64	5.36	4.07	4.46	4.32	4.85
**FDI** ^**b**^	11.55	12.22	8.52	7.73	9.85	9.98
**IPPA Parents_A** ^**c**^	29.48	8.83	29.11	8.15	29.28	8.40
**IPPA Parents_C** ^**d**^	30.83	6.92	32.32	4.31	31.67	5.61
**IPPA Parents_T** ^**e**^	38.73	5.96	40.19	5.37	39.54	5.64
**IPPA Peers_A** ^**f**^	16.68	6.82	16.81	6.11	16.79	6.37
**IPPA Peers_C** ^**g**^	28.13	9.99	28.15	8.13	28.14	8.94
**IPPA Peers_T** ^**h**^	39.71	10.59	41.29	7.94	40.62	9.11
**S-Pan** ^**i**^	11.03	6.56	10.47	6.32	10.72	6.39
**S-Gen** ^**j**^	7.84	3.78	7.45	3.98	7.62	3.87
**S-Sep** ^**k**^	9.39	4.33	8.67	4.08	8.99	4.18
**S-Soc** ^**l**^	8.55	3.34	7.77	3.17	8.11	3.25
**S-Sco** ^**m**^	2.71	2.27	2.27	1.60	2.46	1.92

^**a**^ CSI-24 = Children Somatization Inventory-24

^**b**^ FDI = Functional Disability Inventory

^**c**^ IPPA Parents_A = Alienation factor of the IPPA Parents

^**d**^ IPPA Parents_C = Communication factor of the IPPA Parents

^**e**^ IPPA Parents_T = Trust factor of the IPPA Parents

^**f**^ IPPA Peers_A = Alienation factor of the IPPA Peers

^**g**^ IPPA Peers_C = Communication factor of the IPPA Peers

^**h**^ IPPA Peers_T = Trust factor of the IPPA Peers

^**i**^ S-Pan = SCARED “Panic symptoms/somatic” score

^**j**^ S-Gen = SCARED “Generalized Anxiety” score

^**k**^ S-Sep = SCARED “Separation Anxiety” score

^**l**^ S-Soc = SCARED “Social Anxiety” score

^**m**^ S-Sco = SCARED “School Anxiety” score

### Association between shyness, attachment and somatic symptoms

To further investigate the validity of the Shyness factor that emerged from the CSQ-it, we estimated correlations with convergent and divergent constructs as measured by the SCARED, the CSI-24, the FDI, and the IPPA. A correlational analysis was conducted between the mean values of the age (in months) and gender variables and the total and subscale scores of the entire battery (CSQ-it, SCARED, CSI-24, FDI, IPPA). Overall, gender significantly correlated with the total score of the CSQ-it (r = .167, p < .01), with the “Communication” and “Trust” factors of the IPPA Peers and with the “Separation anxiety” and “Social anxiety” subscales of the SCARED. Age significantly correlated with the total score of both the CSI-24 and the SCARED, with the “Panic”, “Separation anxiety” and “School anxiety” subscales of the SCARED, as well as with the “Communication” and “Trust” factors of the IPPA Peers (see Table 3).

**Table 3:** Correlational analyses.

	1	2	3	4	5	6	7	8	9	12	13	10	11	14	15	16	17
**1 Gender**	1	-.	-														
**2 Age**	-.036	1															
**3 IPPA Parents_A**	-.007	.043	1														
**4 IPPA Parents_C**	.091	.109*	-.072	1													
**5 IPPA Parents_T**	.072	.097*	-.237**	.646**	1												
**6 IPPA Peers_A**	.035	-.035	-.052	.004	-.166**	1											
**7 IPPA Peers_C**	.135**	.101*	-.067	.416**	.347**	-.116*	1										
**8 IPPA Peers_T**	.119*	.102*	-.119*	.384**	.469**	-.208**	.727**	1									
**9 CSQ**	.167**	.011	.104*	.078	-.056	.245**	.030	.032	1								
**10 FDI**	-.014	-.123**	-.046	-.105*	-.151**	.184**	-.100*	-.133**	.235**	1							
**11 CSI-24**	-.036	-.141**	-.014	-.032	-.143**	.292**	-.015	-.087	.210**	.391**	1						
**12 S-Tot.**	.082	-.114*	.055	.059	-.155**	.367**	-.017	-.084	.486**	.318**	.516**	1					
**13 S-Pan**	.050	-.117*	.023	.047	-.167**	.351**	-.024	-.115*	.387**	.307**	.543**	.891**	1				
**14 S-Gen**	.021	-.057	.051	.061	-.129**	.291**	.006	-.059	.361**	.216**	.422**	.848**	.699**	1			
**15 S-Sep**	.115*	-.157**	.059	.070	-.045	.215**	-.004	-.002	.375**	.213**	.322**	.766**	.550**	.540**	1		
**16 S-Soc**	.121**	-.008	.085	.082	-.093*	.274**	-.023	-.047	.546**	.231**	.316**	.783**	.579**	.596**	.535**	1	
**17 S-Sco**	.007	-.119**	-.008	-.094*	-.230**	.372**	-.031	-.126**	.232**	.342**	.434**	.644**	.566**	.503**	.376**	.390**	1

**. p < 0.01.

*. p < 0.05

Note: 1 Gender; 2 Age; 3 IPPA Parents_A = Alienation factor of the IPPA Parents; 4 IPPA Parents_C = Communication factor of the IPPA Parents; 5 IPPA Parents_T = Trust factor of the IPPA Parents; 6 IPPA Peers_A = Alienation factor of the IPPA Peers; 7 IPPA Peers_C = Communication factor of the IPPA Peers; 8 IPPA Peers_T = Trust factor of the IPPA Peers; 9 CSQ = Children Shyness Questionnaire; 10 FDI = Functional Disability Inventory; 11 CSI-24 = Children Somatization Inventory; 12 S-Tot. = SCARED total score; 13 S-Pan = SCARED “Panic symptoms/somatic” score; 14 S-Gen = SCARED “Generalized Anxiety” score; 15 S-Sep = SCARED “Separation Anxiety” score; 16 S-Soc = SCARED “Social Anxiety” score; 17 S-Sco = SCARED “School Anxiety” score

Regarding the relationship between the constructs investigated by the self-report questionnaires, the total score of the CSQ-it correlated significantly with the CSI-24, the FDI and the SCARED total scores. Significant correlations were also found between the CSQ-it and the three factors of the IPPA Peers as well as with the “Communication” factor of the IPPA Parents. The correlation analysis confirmed the divergent validity of the CSQ-it (see Table 3).

## Discussion

To our knowledge, this is the first study to use the CSQ to assess shyness among schoolchildren in Italy. Overall, the findings demonstrated the reliability and validity of the CSQ-it. The goodness of fit of the factorial structure of the CSQ within an Italian context was confirmed, showing the presence of a single factor that defined shyness, in line with previous studies [44,48]. Findings from the confirmatory factor analyses replicated the established single-factor model of this measure previously reported in samples of North American and Chinese children [44,48,66]. Analysis of the factorial structure and psychometric properties of the CSQ-it suggests that internal states such as shyness can be measured through self-report questionnaires since it involves the child’s feelings of self-consciousness and fear of negative self-evaluation. In particular, it suggests that from the age of eight years, children are sufficiently mature to understand and explain their own internal states and are competent in indicating their own levels of shyness.

Moreover, in line with the research hypotheses, higher levels of shyness were associated with anxious and somatic symptomatology, with impaired psychosocial functioning and with specific components of attachment relationships. The negative relationship between shyness and the functioning of schoolchildren also offers support for the second research hypothesis. Consistent with recent studies carried out in the Italian context that have focused on the relationship between shyness, child-teacher relationship and socio-emotional functioning [67], scores on shyness on the CSQ-it correlated with measures of somatic and anxious symptoms. The link between somatic and anxious symptoms may be due to the adoption of a passive coping style, based on social withdrawal and lack of confidence in one's ability to cope with pain. Believing that they cannot deal with pain successfully, children may have recourse to avoidance strategies that limit their daily activities while expanding and prolonging the pain itself, leading to emotional difficulties and disability [68]. Within this frame, the components of shyness play a fundamental role, as confirmed by findings that shy children are more prone to the experience somatic symptoms. Further, despite the fact that shyness is not in itself considered to be a behavioural, social or emotional disorder, its persistence and its severity can be associated with several negative outcomes in children, such as low self-esteem, loneliness and the manifestation of negative affect [66]. Moreover, shy children tend to avoid social situations more than their non-shy peers. As a result, they will have fewer opportunities to learn social skills and are more likely to develop a poor sense of self-efficacy and low self-esteem [69]. Regarding the attachment relationships of shy children, the present findings provide evidence of a link between shyness and the quality of children’s perceived attachment to parents and peers. By experiencing anxious feelings, shy children often prefer to avoid social interactions with peers by limiting their socialization opportunities [24,70]. Consistent with this study’s hypothesis, levels of shyness as measured by the CSQ-it, were associated with the Alienation factor of the IPPA-Peers, emphasizing how secure attachment bonds are connected to greater trust in others, more satisfying interpersonal relationships, and greater peer acceptance [71]. This confirms the difficulty that shy children experience in creating and maintaining relationships with their peers [72]. Similarly, Crozier’s studies [73,74] of the link between shyness and behavioral inhibition found that both shy children and adults are more reticent than their peers in social circumstances, consequently influencing acceptance by their peers. In turn, peer exclusion contributes to maintain or increase social withdrawal over time and to the manifestation and stabilization of shyness. Despite lack of empirical evidence, Booth-LaForce and Oxford [75] have argued that social isolation is linked to insecure attachment showing higher scores on the lack of security in inhibited children. Our results confirm that shy children report greater insecure attachment towards peers and demonstrate greater difficulty in communicating with and trusting peers, as well as higher social alienation.

These findings support the main research goals. However, the present study represents a preliminary step towards validating the CSQ among Italian schoolchildren. As future research, we are currently working on longitudinal study in which we will add future statistical validation adding the test-retest reliability of the CSQ-it.

## Study 2: The moderating role of shyness in the link between somatic symptoms and internet addiction. A study of the discriminant validity of the CSQ-it

Shy children are more likely to develop cognitive and behavioral patterns that make them vulnerable to addiction to the internet, even if not all shy children use the internet in an excessive or risky way, and not all those who use the internet excessively experience somatic symptomatology. The aim of the second study is to test the hypothesis that shyness moderates the link between somatic symptoms and internet addiction and to explore the specific role of somatic symptoms in the association between shyness and internet addiction, hypothesizing a mediating effect.

In moderation analyses, the interaction between variables occur when the effect of an independent variable on a dependent variable varies across levels of a moderating variable. Specifically, in the present study somatic symptoms represent the independent variable, internet addiction is the dependent variable and shyness is defined as moderating variable. This model is chosen in order to explore the role of shyness in the association between the presence of a somatic symptomatology and addiction to the internet among adolescents.

Mediation analyses were carry out for testing hypothetical processes and mechanisms through which an independent variable might have an indirect effect over a dependent outcome variable through a mediators. A mediational analysis was conducted to examine the direct effect of shyness on internet addiction, and its indirect effect through somatic symptoms as measured by the CSI-24 on addiction to the internet.

## Method

### Participants

The study involved a sample of 131 schoolchildren attending the first grade of an Italian school (Males N = 64, 48.9%; and Females N = 67, 51.1%). Participants ranged in age from 10 to 15 years (M = 12.50; SD = 0.99). Twenty-one participants provided more than 10% of missing values and therefore, were excluded from the analysis. The final sample therefore comprised 110 schoolchildren with an average age of M = 12.17 (SD = 0.98; Males N = 54, 49.1%). All participants were Caucasian.

### Procedure

A written informed consent was obtained from all schoolchildren and their parents before their enrolment in the study. All participants included in the final sample (N = 110) completed the entire questionnaire battery that was administrated in the classroom during school time. The collective administration took approximately 20–30 minutes. Anonymity of participants was ensured. This study was approved by the Ethics Committee of the Department of Dynamic and Clinical Psychology, Sapienza University of Rome.

### Measures

#### Shyness

The Italian version of the Children’s Shyness Questionnaire (CSQ) [44] was used to assess shyness the participants. In this study the Cronbach alpha was.72.

#### Somatic symptoms

As in study 1, the Children’s Somatization Inventory-24 Child Version (CSI-24) [54,55] assessed children’s perceptions of somatic symptoms. In the current study Cronbach alpha was .81.

#### Internet addiction

The Internet Addiction Test (IAT) [76] was designed to assess the use of the internet and the severity of symptoms of overuse among children and adolescents. The IAT is a self-report questionnaire consisting of 20 items rated on a six-point Likert scale (0 “Does not apply”; 1 “Rarely”; 2 “Occasionally”; 3 “Frequently”; 4 “Often” and 5 “Always”). The total score of the scale ranges from 0 to 100. Higher scores indicate a greater level of addiction and problems caused by internet usage. Although researchers have used different cut-off points for internet addiction, a clinical or empirical cut-off for the IAT has yet to be validated [77]. In previous studies, the IAT has consistently demonstrated high reliability with adolescent samples (Cronbach’s α > .80) [78].In the current study Cronbach alpha was .86. For the purpose of the present study we used the average score of internet addiction.

## Results

The mean Shyness score was 13.6 (SD = 7.89) with females (M = 15.5, SD = 8.54) scoring significantly (F(1,108) = 6.98, p < 0.01, η^2^ = 0.061) higher on average than males (M = 11.63, SD = 6.68). Considering the cut-off scores of Shyness, we identified 11 adolescents with Shyness scores greater than 1 SD above the mean. Turning to somatization, we found that approximately 50% of participants reported no somatic complaints, 12.7% reported only one symptom (n = 14), 20.1% reported two to four symptoms (n = 22), 16.5% complained of five to eight symptoms (n = 16) and 2.7% reported more than eight symptoms (n = 3). Considering internet addiction, the sample’s IAT score was M = 43.66 (SD = 12.21) with males scoring somewhat higher (M = 44.67; SD = 12.41) than females (M = 42.70, SD = 12.05), although the difference was not statistically significant (F(1,108) = 0.714, df = 1, p = 0.400, η^2^ = 0.006).

Correlational analyses showed a significant association between CSQ-it and gender (r = .246, p < 0.01), age (r = .245, p = 0.01) and CSI-24 (r = .296, p < .01). In addition, CSI-24 scores significantly correlated with the IAT scores (r = 0.275, p < 0.01).

### Moderated mediation model

**Fig 1:**
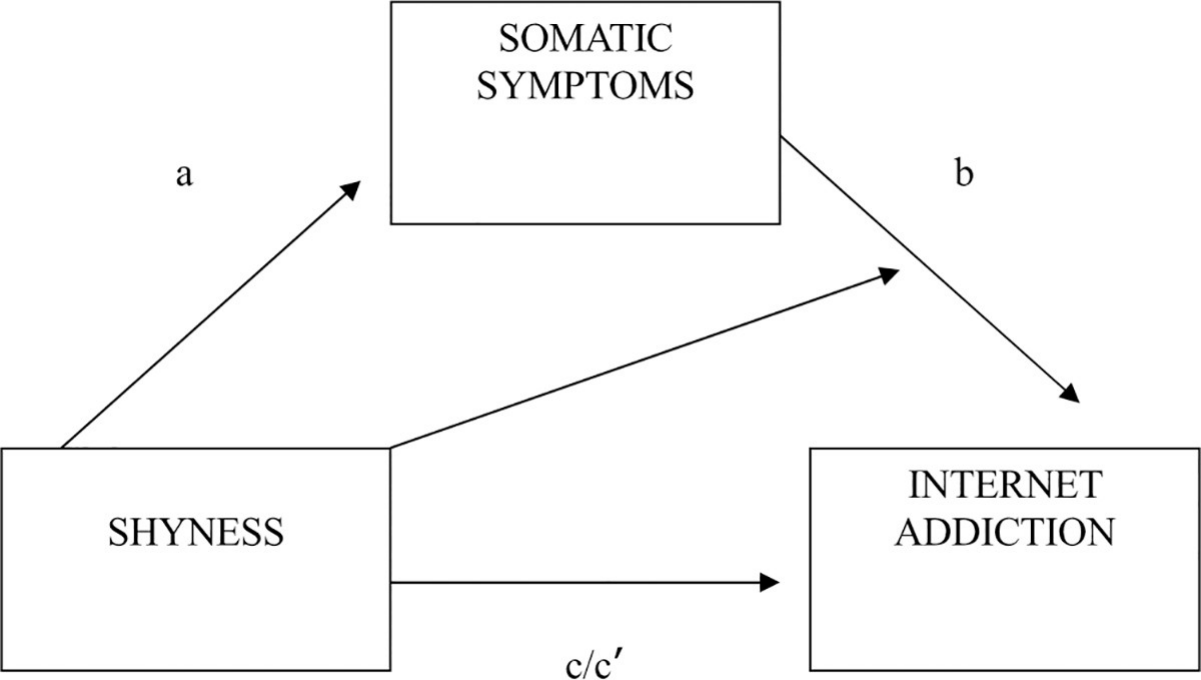
Moderated mediation model.

To test the hypothesis that the relationship between shyness and internet addiction is mediated by somatic symptoms and that shyness moderates the relationship between somatic symptoms and internet addiction we tested a moderated mediation model (Fig 1) using the macro PROCESS by Hayes [79].

Results suggested that shyness predicts an increase in somatic symptoms (β = 0.51, SE = .15, p < 0.01) and somatic symptoms predict an increase in internet addiction (β = .26, SE = .09, p < 0.01). However, shyness is not related to an increase in internet addiction (β = .03, p = 0.85) but the interaction between shyness and somatic symptoms is positively related to increased internet addiction scores (β = .03, SE = .01, p = .01). Also the index of moderated mediation was significant (index = .0148, boot-SE = .0056, bootstrap 95% C.I.: .0051: .0278). More importantly, the indirect effect is significantly moderated for high shyness scores (β = .26. boot-SE = .08, bootstrap 95% C.I: .1186; .4437) but not for low levels of shyness (β = .03, boot-SE = .08, bootstrap 95% C.I: -.0911; .2400). In summary, high levels of shyness are associated with somatic symptoms which subsequently are associated with increased risk of internet addiction.

## Discussion

The aim of this second study was to explore the link between shyness, internalizing problems and internet addiction. Before testing the hypotheses underlying the study, the first step provided further evidence of the sound psychometric properties of the CSQ-it in terms of divergent validity in the Italian context. Second, the role of somatic symptoms in the relationship between shyness and internet addiction was investigated and the interaction between shyness and somatic symptoms was evaluated in predicting internet addiction.

Adolescence is a stage characterized by profound changes, both emotionally and behaviorally, in which the body plays a central role. Somatic symptomatology refers to physical symptoms whose genesis and evolution mainly depend on psychological factors. High levels of shyness, especially when they are associated with social withdrawal, can lead to loneliness and isolation, contributing to the manifestation of emotional and somatic problems [9]. The results presented here show a significant correlation between shyness and somatic symptoms. A possible explanation for this link might be that children with higher levels of shyness may experience greater inner discomfort related to social situations, in terms of anxiety and nervousness, and tend to express their discomfort through their body. It is well-documented that those who feel worried, anxious or tense, respond to external demands with the activation of reactions that may lead to pathological outcomes if the discomfort persists over time [80].

Several studies have explored the role of shyness in the expression of somatic symptomatology, but none has yet examined the relationship between these two constructs in the context of risk of internet addiction. Our findings are consistent with the study hypotheses. The analysis of the models of mediation and moderation indicates that, when the effect of somatic symptoms is controlled, there is a significant link between shyness and internet addiction. However, shyness alone does not predict internet addiction, since the direct path is not significant. In contrast, a significant indirect path supported the hypothesis of greater impaired adaptive functioning if shyness presents a somatic symptomatic picture.

During social interactions, children with high levels of shyness continually experience fear of making mistakes and dysfunctional thoughts, contributing to generating anxiety that may lead to a tendency to avoid potential threatening situations [81]. Therefore, in view of the problems shy children associate with a face-to-face relational context, their online interactions are worthy of investigation. In particular, the role of shyness in moderating the relationship between somatic symptoms and internet addiction was examined. Several studies have focused on how the use of the internet may improve an individual’s social relationships [82]. However, while the internet is one of the most accessible forms of media in the world, potential adverse effects are linked to its problematic use [49], particularly when it replaces “real life” with an online life [82].

The main risk is that the internet, rather than being one aspect of adolescent life, becomes the central part of an adolescent’s existence, with several repercussions in dealing with reality, including relational problems. These aspects assume an even greater value for individuals characterized by excessive levels of shyness, as confirmed by the present study wherein it was shown that higher levels of shyness are associated with an increase in the risk of developing an internet addiction. The internet assumes a compensatory function with respect to unsatisfactory relationships. Findings from this second study emphasize the role of shyness as a moderator. Several studies have investigated moderating variables between shyness and different aspects of mental health, such as eating disorders [83], depression [84], and somatic symptoms [11]. It is probable that in the presence of somatic symptomatology, shyness represents a risk factor for problematic internet use, in the sense that it significantly increases the likelihood that a person will experience a certain disorder in a short time and in a different way than when this factor is not present.

## Conclusion

The primary goal of the studies presented here was to broaden our knowledge of shyness in an Italian sample by providing new empirical evidence about its measurement and its correlates.

Both studies demonstrate the reliability and validity of the CSQ-it, suggesting its value as a sensitive and appropriate instrument for the assessment of shyness during childhood and adolescence. The exploratory and confirmatory analyses confirm the good fit of the factorial structure of the questionnaire, allowing, at the same time, the validation of a single factor to define shyness within an Italian context. Furthermore, the analysis of the factorial structure and the psychometric properties of the CSQ-it indicate the possibility of effectively measuring internal states, such as shyness, through self-report questionnaires in school age children. These results provide evidence of the impact of shyness on psychosocial functioning and on the quality of meaningful relationships with parents and peers, as well as on the role that it plays in communication and social interactions.

However, our findings should be interpreted while keeping its limitations in mind. First, our sample consisted of healthy children, so it is not clear whether the results may be generalized to other populations (e.g., with chronic physical illnesses or pain). Second, this was a cross-sectional study and, consequently, the conclusions drawn should be considered with caution. In particular, cross-sectional studies do not provide the opportunity to demonstrate a temporal relationship, limiting the ability to infer causation. Only with longitudinal data will it be possible to establish a true cause and effect relationship and this is important for future research. Furthermore, we carried out a mediational analyses although the cross-sectional design to mediation can produce substantially biased estimates of longitudinal parameters even when the mediation is complete. Third, the Functional Disability Inventory was translated but not validated in Italian culture. Finally, our data were based on self-report measures rather than objective assessment, and susceptible to respondent bias.
